# IrrE, a Global Regulator of Extreme Radiation Resistance in *Deinococcus radiodurans*, Enhances Salt Tolerance in *Escherichia coli* and *Brassica napus*

**DOI:** 10.1371/journal.pone.0004422

**Published:** 2009-02-10

**Authors:** Jie Pan, Jin Wang, Zhengfu Zhou, Yongliang Yan, Wei Zhang, Wei Lu, Shuzhen Ping, Qilin Dai, Menglong Yuan, Bin Feng, Xiaoguang Hou, Ying Zhang, Ruiqiang, Tingting Liu, Lu Feng, Lei Wang, Ming Chen, Min Lin

**Affiliations:** 1 Biotechnology Research Institute, Chinese Academy of Agricultural Sciences, Key Laboratory of Crop Biotechnology, Ministry of Agriculture, Beijing, China; 2 Key Laboratory for Nuclear Waste Treatment and Environmental Safety, Commission of Science, Technology and Industry for National Defence, Southwest University of Science and Technology, Mianyang, China; 3 National Centre for Plant Gene Research, Beijing, China; 4 TEDA School of Biological Sciences and Biotechnology and Tianjin Key Laboratory of Microbial Functional Genomics, Nankai University, Tianjin, China; Baylor College of Medicine, United States of America

## Abstract

**Background:**

Globally, about 20% of cultivated land is now affected by salinity. Salt tolerance is a trait of importance to all crops in saline soils. Previous efforts to improve salt tolerance in crop plants have met with only limited success. Bacteria of the genus *Deinococcus* are known for their ability to survive highly stressful conditions, and therefore possess a unique pool of genes conferring extreme resistance. In *Deinococcus radiodurans*, the *irrE* gene encodes a global regulator responsible for extreme radioresistance.

**Methodology/Principal Findings:**

Using plate assays, we showed that IrrE protected *E. coli* cells against salt shock and other abiotic stresses such as oxidative, osmotic and thermal shocks. Comparative proteomic analysis revealed that IrrE functions as a switch to regulate different sets of proteins such as stress responsive proteins, protein kinases, glycerol-degrading enzymes, detoxification proteins, and growth-related proteins in *E. coli*. We also used quantitative RT-PCR to investigate expression of nine selected stress-responsive genes in transgenic and wild-type *Brassica napus* plants. Transgenic *B. napus* plants expressing the IrrE protein can tolerate 350 mM NaCl, a concentration that inhibits the growth of almost all crop plants.

**Conclusions:**

Expression of IrrE, a global regulator for extreme radiation resistance in *D. radiodurans*, confers significantly enhanced salt tolerance in both *E. coli* and *B. napus*. We thus propose that the *irrE* gene might be used as a potentially promising transgene to improve abiotic stress tolerances in crop plants.

## Introduction

Soil salinity is one of the major abiotic stresses leading to depression of crop yields [1]. This problem is becoming more severe because of soil degradation, water shortage and global warming. Clearly, the development of transgenic crops that can tolerate high salt stress would offer a practical contribution to solving this urgent problem. Considerable efforts have been made to increase the salt tolerance of crops, not only by exploitation of natural genetic variation, but also by transferring foreign genes into crops [2], [3]. Genes used in the transgenic approach have included those encoding functional and regulatory proteins [4], [5]. Functional proteins, including enzymes required for biosynthesis of various osmoprotectants, ion transporters for maintaining high K^+^ and low Na^+^ homeostasis and detoxification enzymes, directly protect against environmental stresses. Regulatory proteins were shown to be involved in control of gene expression and signal transduction in response to multiple stresses. They include transcription factors, protein kinases and enzymes involved in phosphoinositide metabolism. However, due to the fact that salt tolerance is a complex trait and that the underlying molecular mechanisms are not well-understood, such strategies have met with only limited success [6]. The discovery of genes involved in various stress responses provides new targets for improvement of stress tolerance in crop plants.

The genus *Deinococcus*, which was first isolated in 1956 [7], comprises more than twenty distinct species that can survive acute exposure to ionizing radiation (10 kGy) and ultraviolet light (1 kJ/m^2^), as well as longer-term exposure to desiccation and IR (60 Gy/h) [8]. Bacteria of this genus, known for their ability to survive extreme stress conditions, possess a unique pool of genes conferring extreme resistance [9]. Until now, complete genome sequencing has been established for *D. radiodurans* R1 and *D. geothermalis* [9], [10]. Genetic analysis of a DNA damage-sensitive strain of *D. radiodurans* R1 led to the discovery of a novel regulatory protein (DR0167, also named PprI) [11], [12]. The IrrE protein can stimulate transcription of *recA* and *pprA*, which encode recombinase A and the radiation-inducible protein following irradiation [12]. Interestingly, constitutive expression of the irrE gene in *E. coli* using a shuttle plasmid under the control of a GroESL promoter promotes DNA repair and offers oxidative damage protection [13]. *E. coli*, as a representative strain of prokaryotes, is of interest industrially, genetically and pathologically [14]–[16]. The response to salt stress has been extensively studied in *E. coli* [17], [18]. This model organism may therefore be well-suited to investigating regulation by IrrE. *B. napus* is one of the most important oilseed crops cultivated worldwide, and it is sensitive to salt stress throughout the growing season. Transgenic *B. napus* plants overexpressing AtNHX1, a vacuolar Na^+^/H^+^ antiporter from *Arabidopsis thaliana*, were able to grow, flower, and produce seeds in the presence of 200 mM NaCl [19], suggesting the possibility of engineering crop plants with improved salt tolerance. The purpose of the present work is to investigate whether the *irrE* gene can be utilized to improve tolerance to other abiotic stresses and, in particular, tolerance to high salinity. We demonstrated here that expression of IrrE, a global regulator for extreme radiation resistance in *Deinococcus radiodurans*, confers significantly enhanced salt tolerance in both *E. coli* and *Brassica napus*. Our data suggest that IrrE acts as a switch to control a set of genes involved in different metabolic and signaling pathways, leading to dramatically enhance salt tolerance in these genetically modified organisms.

## Results

### IrrE protects *E. coli* cells against various abiotic stresses

**Figure 1:**
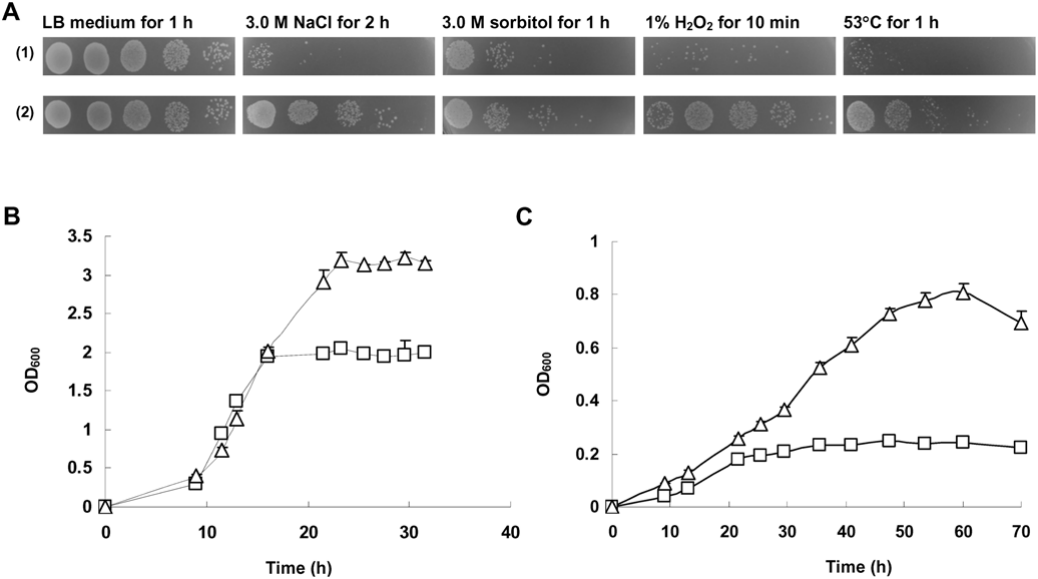
Effects of abiotic stresses on growth of *E. coli* strains. A: Growth of the *E. coli* control strain carrying only the pMG1 vector (1) and the transformant strain expressing IrrE (2) in LB medium after exposure to various abiotic stresses; B: Growth of the control strain (squares) and the IrrE-expressing strain (triangles) in M9 minimal medium; C: Growth of the control strain and the IrrE-expressing strain in M9 minimal medium containing 0.65 M NaCl.

To study the effect of IrrE in *E. coli*, we compared the stress response with an *E. coli* control strain carrying only the pMG1 vector and a transformant strain expressing IrrE. Using LB plate assays, as shown in **Figure 1A**, IrrE protected *E. coli* cells against salt shock and other abiotic stresses such as oxidative, osmotic and thermal shocks. The effect of salt stress on the growth of control strain and IrrE-expressing strain was examined in M9 minimal medium. When the IrrE-expressing strain was inoculated into M9 minimal medium, it also displayed better growth than the control strain with higher maximal cell density (**Figure 1B**). In the presence of 0.65 M NaCl, the IrrE-expressing strain reached a maximum OD_600_ of 0.88 after 60 h of incubation, while the control strain displayed significantly impaired growth (**Figure 1C**).

### IrrE-overexpressing transgenic plants display significantly improved salt tolerance

**Figure 2:**
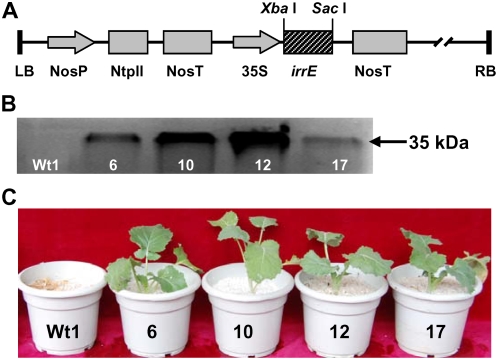
Overexpression of IrrE improves salt tolerance in *B. napus* plants. (A) Diagram of the P_35S_:IrrE construct; (B) Western blot analysis of IrrE in wildtype plant (Wt1) and transgenic lines no.6, 10, 12, 17. Molecular mass is indicated on the right; (C) Salt treatment of Wt1 and representative transgenic plants. After 350mM NaCl treatment for six weeks, representative plants were photographed.

To assay the effect of IrrE expression on salt tolerance in a crop plant, we generated *Brassica napus* transgenic plants overexpressing the *irrE* gene (**Figure 2**). As shown in **Figure 2A**, a construct containing the full-length cDNA of the *irrE* gene under the control of the cauliflower mosaic virus 35S promoter was introduced into the genome of *Brassica napus* cultivar Shuanzha no.9 using an *Agrobacterium*-mediated transformation method. Following two rounds of kanamycin selection, 126 kanamycin resistant plants were regenerated. PCR analysis revealed that 42 plants showed amplification of the predicted *irrE* gene fragment. Seven homozygous lines from these transgenic plants were obtained in the T2 generation. Southern blot analysis suggested that seven transgenic lines had one or more copies of the *irrE* gene (data not shown). Western blot analysis confirmed the expression of IrrE in four independent transgenic lines, but not in the wild-type control plants (**Figure 2B**). The expression levels of IrrE vary among the different lines, which could be attributed to different integration sites of the transgene or different transgene copy number [20].

Two weeks after germination, four independent transgenic lines of *B. napus* and wild-type controls were treated with 350 mM NaCl for six weeks. All transgenic plants grew well in th.e presence of 350 mM NaCl, whereas growth of wild-type plants was significantly inhibited and the plants died two weeks thereafter under these conditions (**Figure 2C**). Transgenic plants treated with 350 mM NaCl were able to flower and produce seeds normally when transferred into the field. No significant difference was detected between salt-treated transgenic plants and control plants that had not been exposed to salt stress, as evaluated by various agronomic traits such as plant height, length of main inflorescence, number of pods per plant, number of seeds per pod and weight of 1,000 seeds (data not shown).

### Protein profile analysis of the IrrE-expressing strain in response to salt shock

**Figure 3:**
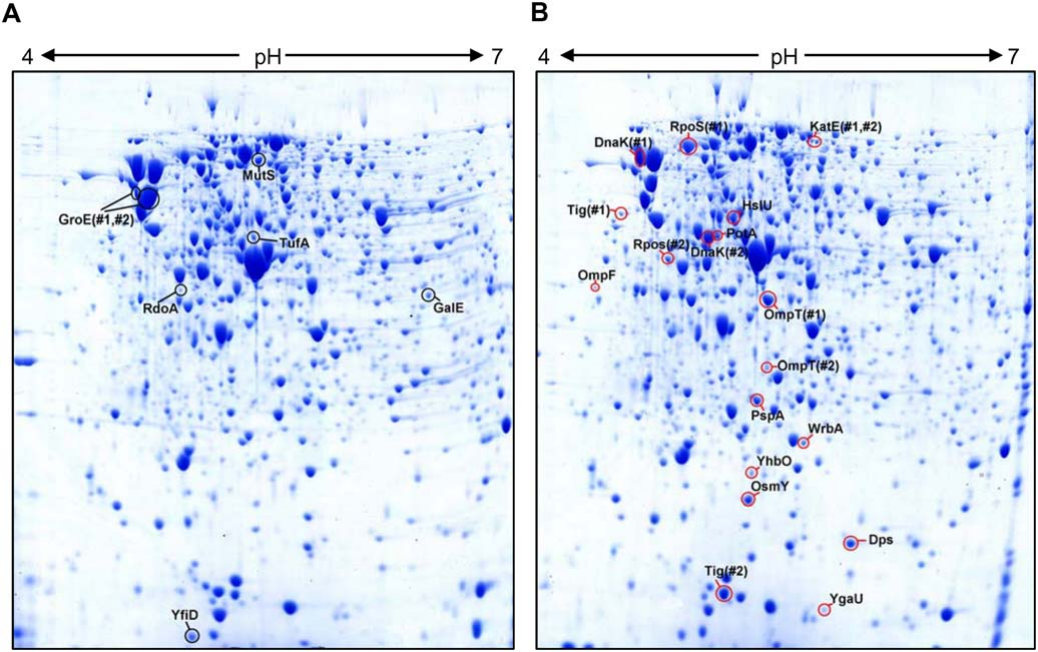
Protein profiles of *E. coli* control strain (A) and IrrE-expressing strain (B) in response to salt shock. Previously characterized stress-inducible proteins with increased abundance after NaCl shock in the IrrE-expressing strain (red boxes) or the control strain (black boxes) are marked. At least six replicated gels from two independent experiments were analyzed and spots reproduced in all replicates were excised for MALDI-TOF MS analysis. Multispot proteins are numbered. MW, molecular weight, in thousands.

**Figure 4:**
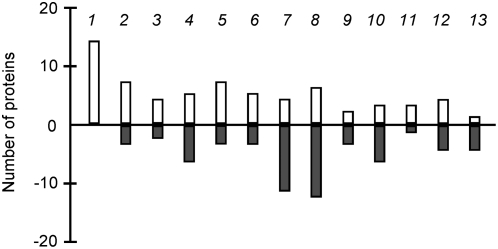
Functional profiling of up- and downregulated proteins in the IrrE-expressing *E. coli* strain compared with the control strain. The functional role category annotation is that provided by BioCyc (http://biocyc.org/). Each column represents the number of proteins with at least twofold changes in a selected functional category. Positive and negative values indicate up- and down-regulation, respectively. Columns: 1, nucleotide biosynthesis; 2, amino acid biosynthesis; 3, biosynthesis of cofactors, small molecule carriers; 4, RNA synthesis, modification, DNA transcription; 5, proteins (translation and modification); 6, proteins (chaperones); 7, energy metabolism; 8, carbon utilization; 9, central intermediary metabolism; 10, transport and binding proteins; 11, cytoskeleton; 12, cellular processes and 13, hypothetical proteins.

**Figure 5:**
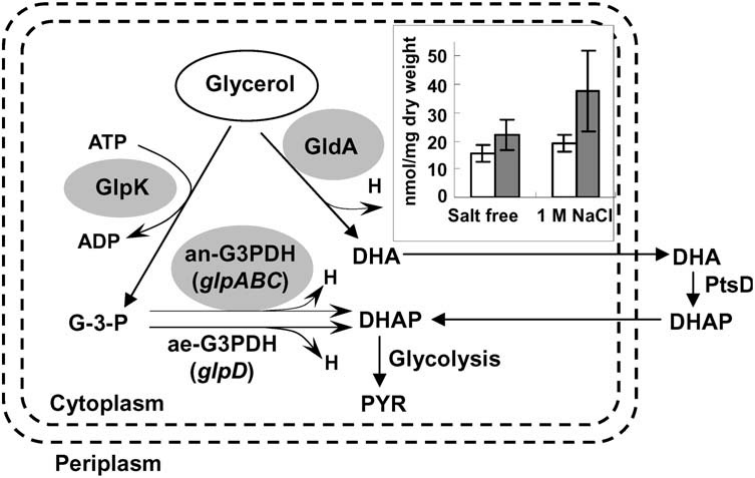
Schematic representation of the *E. coli* glycerol dissimilation pathways and the change in intracellular glycerol content. Expression levels of the major enzymes were found to be decreased by at least 2-fold (grey shading), as indicated by proteomics analysis. Inset shows the change in intracellular glycerol content in an IrrE-expressing strain (▪) and a control strain (□) after incubation for 1 h with or without 1.0 M NaCl. Data represent the means and standard errors of three replicate experiments in which each sample was assayed in duplicate. Statistical significance was determined by Student's t test (n = 6, P<0.05). Abbreviations: GlpK, ATP-dependent glycerol kinase; GldA, Glycerol dehydrognease; ae-G3PDH, aerobic G3PDH; an-G3PDH, anaerobic G3PDH; PtsD, DHA-specific enzyme II; H, reducing equivalents (H = NADH/NADPH); GPD, glycerophosphoryl diester; DHA, dihydroxyacetone; DHAP, dihydroxyacetone phosphate; PYR, pyruvate.

We further performed experiments to investigate the underlying molecular mechanisms of the enhanced global stress responses in IrrE-expressing *E. coli* and *B. napus*. We first used two-dimensional protein gels to compare global changes in the proteome of the IrrE-expressing *E. coli* strain and the control strain carrying only the pMG1vector. Both strains were subjected to high salt shock and the proteins were extracted and subjected to two-dimensional gel electrophoresis analysis (**Figure 3**). Using a cutoff criterion of 2.0-fold change and 95% confidence, we detected significant changes for 124 proteins, with 66 proteins being upregulated in the IrrE-expressing strain. These up- or downregulated proteins can be grouped into 13 classes based on their common functional characteristics, indicating that IrrE plays a global regulatory role in gene expression in *E. coli* (**Figure 4** **,** **Table S1** **,** **S2**). Among these 13 classes, five major groups might contribute in various ways to the enhanced salt resistance: **(i) Stress responsive proteins.** RpoS is known to be a fundamental regulator of general stress responses to various stress conditions in *E. coli* [17], [18], [21], [22]. We found significant induction of RpoS, which exhibits a 3-fold-higher expression in the IrrE-expressing strain than in the control strain. Furthermore, accumulation of a set of proteins usually induced by environmental stress was observed in the IrrE-expressing strain, including the molecular chaperone DnaK, the heat shock protein HslU and the osmotically inducible protein OsmY **(** **Figure 3** **)**. In addition, about twenty other proteins not detectable in the control strain were induced in the IrrE-expressing strain **(** **Table S1** **)**, including the general stress protein YhbO required for the protection of bacterial cells against many environmental stresses [23], the trigger factor Tig involved in protein export as a chaperone, and the stress-inducible ATP-dependent protease Lon which is induced under stress conditions such as high temperatures [16]. **(ii) Protein kinases.** In microorganisms and plants, protein kinases generally act as sensory molecules in response to environmental changes. We found nine protein kinases that were up- or downregulated with a ratio exceeding two in the IrrE*-*expressing strain compared to the control strain. Expression of nucleoside diphosphate kinase Ndk, which is responsible for maintaining a pool of nucleotide triphosphates for the synthesis of DNA and RNA in *E. coli* [24], was found to be upregulated by about 3-fold in the IrrE-expressing strain in response to salt shock; **(iii) Glycerol-degrading enzymes.** None of the levels of enzymes involved in glycerol biosynthesis showed obvious changes. The respiratory metabolism of glycerol in *E. coli* is mediated by ATP-dependent glycerol kinase GlpK and aerobic/anaerobic respiratory glycerol-3-phosphate dehydrogenases GlpD/GlpABC [25]. Glycerol dehydrognease (GldA) catalyzes the oxidation of glycerol to DHA [26]. These key enzymes, GlpK, GlpA, GlpB and GldA, were downregulated after salt shock, which might lead to the increased accumulation of glycerol in the IrrE-expressing strain. We examined glycerol levels in an IrrE-expressing strain and a control strain subjected to salt treatment. The glycerol level in IrrE-expressing cells reached 37 nmol/mg dry weight and was approximately 2-fold higher (P<0.05) than that in control cells (**Figure 5**). Accumulation of high levels of glycerol, a major feature of osmoregulation in fungi [27], could lead to improved tolerance to various abiotic stresses; **(iv) Detoxification proteins.** Bacteria and plants employ various detoxifying enzymes, such as superoxide dismutase and catalase, to scavenge reactive oxygen species (ROS) generated by salt stress [28], [29]. We found significant induction of detoxification proteins, including a catalase HPII (KatE), which serves to protect cells from the toxic effects of hydrogen peroxide [30], and a tryptophan repressor binding protein (WrbA) which could play a role in the oxidative stress response of diverse prokaryotes [31]. **(v) Metabolism or growth-related proteins.** A number of metabolic enzymes exhibited twofold differential expression in the IrrE-expressing strain compared to the control strain, including those involved in glycolysis, the TCA cycle and the pentose phosphate pathway. The largest proportions of upregulated proteins were related to biosynthesis of nucleotides. Expression of RpsF, a 30S ribosomal subunit protein S6, whose level of expression always correlates with increasing growth rate [16], [32], increased up to 36-fold in the strain expressing IrrE. These results suggested that IrrE could execute a global regulatory role on gene expression in *E. coli* and the altered metabolism or signaling events lead to enhanced stress resistance.

### Stress-responsive gene expression analysis in transgenic *B. napus* plants

**Figure 6:**
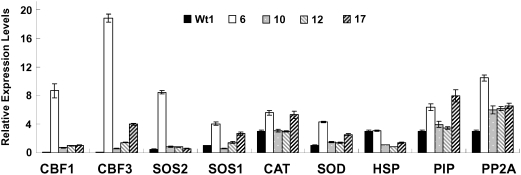
Quantitative RT-PCR analysis of relative expression level of nine selected stress-responsive genes in transgenic and wild-type *B. napus* plants. Abbreviations: CBF1/CBF3, CBF-like protein; SOS2, serine/threonine protein kinase; SOS1, the plasma membrane Na^+^/H^+^ antiporter; CAT, catalase; SOD, manganese superoxide dismutase; HSP, heat shock cognate protein Hsc70; PIP, the plasma membrane intrinsic proteins PIPs; and PP2A, phosphatase 1.

To study the mechanism that confers salt tolerance in transgenic *B. napus* plants overexpressing IrrE, we used quantitative RT-PCR to investigate expression of nine selected stress-responsive genes in transgenic and wild-type *B. napus* plants **(** **Figure 6** **)**. We found that genes encoding pekinensis CBF-like protein CBF1/CBF3, serine/threonine protein kinase SOS2, the plasma membrane Na^+^/H^+^ antiporter SOS1 and manganese superoxide dismutase SOD were upregulated in the transgenic plants. Strikingly, although accumulation of the IrrE protein in transgenic line no. 6 was lower than other lines, expression of most of these selected stress-inducible genes, especially *CBF1* and *CBF3*, was much higher in this line, the reason for which is currently unknown. The differences in transgene expression between these independent transgenic lines could be due to the positional effect or different copy number of the transgene [20]. Consequently, the differential expression of the transgene could cause variations in gene expression pattern among the different transgenic lines, as observed in this study. Additionally, the functional categories of these selected genes may provide important clues to the regulatory mechanisms of IrrE-conferred salt tolerance in transgenic plants. The upregulated genes seem to be involved in at least three pathways: (i) transcriptional regulation of stress-inducible genes by CBF1/CBF3; (ii) the SOS signaling pathway for ion homeostasis under salt stress; and (iii) ROS scavenge by detoxifying enzymes. The altered stress-responsive genes and signaling pathways may collectively contributed to the enhanced salt tolerance in the transgenic *B. napus* plants. Further studies are required to elucidate the molecular link between IrrE expression and changes in expression of these selected genes.

## Discussion

Abiotic stress tolerance is known to be a complex trait. Microarray studies revealed that at least several hundred genes are involved in response to abiotic stress in plants [33]. Many efforts have been made to increase tolerance to drought, salinity, and low temperature in *Arabidopsis* and other plant species by expression of stress-inducible transcription factors [34], [35], [36]. For example, Karaba *et al* reported that expression of the *HARDY* gene encoding an AP2/ERF-like transcription factor from *Arabidopsis* enhanced stress tolerance in rice [37]. It was proposed that a combination of transcription factor that act in different pathways such as ROS scavenging and osmotic adaptation, may prove even more beneficial for improving stress tolerance [5]. However, progress in successful genetic engineering of salt-tolerant crops has been limited by our lack of understanding of the molecular basis of salt tolerance and limited choices of effective genes that confer salt resistance. In some instances, transgenic plants expressing the stress-responsive genes exhibited abnormal growth and development, which adversely affected some important agronomic traits. For example, use of the strong constitutive 35S promoter to drive overexpression of DREB1A resulted in severe growth retardation under normal growing conditions [34].

Microbial sources have recently received much attention because of their exceptionally high diversity. It has been previously proposed that there are remarkable similarities between bacteria and plants in their cellular responses to osmotic stress, because organisms from both kingdoms accumulate the same set of cytoplasmic solutes upon exposure to conditions of hyperosmolarity. Thus, closely parallel mechanisms likely exist in bacteria and plants to mediate responses to various stresses [38]. Efforts have been made to engineer stress-tolerant crops using microbial genes, such as *bet*-cluster genes encoding the choline oxidation enzymes CDH and betaine aldehyde dehydrogenase from *E. coli* [39], the *HAL1* gene encoding a potent modulator of ion homeostasis in yeast [40], a Na^+^/H^+^ antiporter gene from halotolerant cyanobacterium *Aphanothece halophytica* [41], a mitogen-activated protein kinase gene from the Dead Sea fungus *E. herbariorum* [42] and a cold-shock protein from *E. coli* [43]. The ability of transgenic *B. napus* expressing IrrE to tolerate 350 mM NaCl further substantiated the potential value of microbial genes for improving plant agronomic traits and suggests that IrrE could be a potentially effective target gene for engineering salt tolerant crop plants. To our knowledge, this is the first report of salt stress tolerance attributable to expression of a bacterial global regulator in a crop plant.

**Figure 7:**
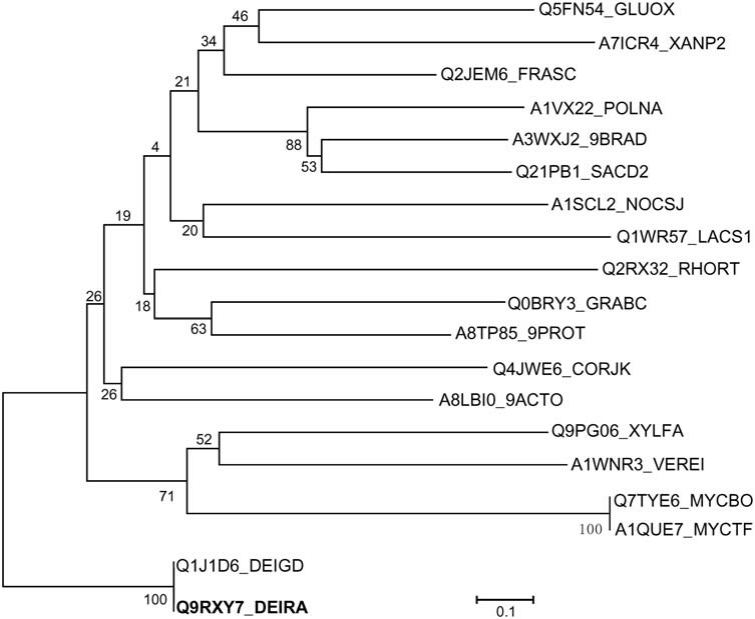
Unrooted neighbour-joining phylogenetic tree from the alignment of the *D. radiodurans* R1 IrrE protein with related proteins. Bootstrap values based on 1000 replications are listed as percentages at branching point. Bar, 0.1 substitutions per position. The sequence abbreviations, species and functional descriptions are as follows: Q9RXY7_DEIRA, *Deinococcus radiodurans* R1, IrrE; Q1J1D6_DEIGD, *Deinococcus geothermalis* DSM 11300, Zn dependent hydrolase fused to HTH domain, IrrE ortholog; Q5FN54_GLUOX, *Gluconobacter oxydans*, Transcriptional regulator; Q9PG06_XYLFA, *Xylella fastidiosa*, Putative uncharacterized protein; Q0BRY3_GRABC, *Granulibacter bethesdensis* ATCC BAA-1260, Putative uncharacterized protein; A1VX22_POLNA, *Polaromonas naphthalenivorans* CJ2, Putative uncharacterized protein; A7ICR4_XANP2, *Xanthobacter autotrophicus* ATCC BAA-1158, Putative uncharacterized protein; Q2JEM6_FRASC, *Frankia sp.* CcI3, Putative uncharacterized protein; A1SCL2_NOCSJ, *Nocardioides sp.* BAA-499, Helix-turn-helix domain protein; A8TP85_9PROT, *Alpha proteobacterium* BAL199, Putative uncharacterized protein; A1WNR3_VEREI, *Verminephrobacter eiseniae* EF01-2, Putative uncharacterized protein; Q1WR57_LACS1, *Lactobacillus salivarius subsp. salivarius* UCC118, Transcriptional regulator; Q2RX32_RHORT, *Rhodospirillum rubrum* ATCC 11170, Putative uncharacterized protein; A3WXJ2_9BRAD, *Nitrobacter sp.* Nb-311A, Putative uncharacterized protein; Q4JWE6_CORJK, *Corynebacterium jeikeium* K411, Putative uncharacterized protein; Q21PB1_SACD2, *Saccharophagus degradans* ATCC 43961, Putative uncharacterized protein; Q7TYE6_MYCBO, *Mycobacterium bovis*, Putative uncharacterized protein Mb2544c; A1QUE7_MYCTF, *Mycobacterium tuberculosis* F11, Putative uncharacterized protein; A8LBI0_9ACTO, *Frankia sp.* EAN1pec, Putative uncharacterized protein.

IrrE is believed to function as a transcriptional regulator that is required for *D. radiodurans* resistance to ionizing radiation, UV light, and mitomycin C. Since its identification from *D. radiodurans* in 2002, up to now, still relatively little is known about the mechanism of IrrE action in *D. radiodurans*. The IrrE protein contains an Xre-like HTH domain and a zinc-dependent protease domain, but no sequences recognized by IrrE have been reported yet [9]. Furthermore, purified IrrE protein does not directly bind to the promoter region of *recA* or other induced genes [44]. Further, the *D. radiodurans* IrrE homologue is detected only in *D. geothermalis* based on genomic database searches and phylogenetic analysis, suggesting that IrrE appears to be a *Deinococcus* genus-specific regulator (**Figure 7**). Thus, it is a highly intriguing question with regard to how the IrrE protein functions in the heterologous *E. coli* and *B. napus* systems to confer enhanced stress tolerance. In fact, similar phenomena have also been observed in pathogen-host plant interactions. For example, plant pathogenic *Pseudomonas syringae* deliver bacterial virulence factor proteins into the host cell, where they function to manipulate host defense and metabolism to benefit the extracellular bacterial colony [45], [46]. Also, *Agrobacterium* is well known for its ability to hijack fundamental cellular processes during the genetic transformation of its hosts. In a recent paper, Djamei *et al*. showed that VirE2-interacting protein 1 is a transcription factor that is a direct target of the *Agrobacterium*-induced mitogen-activated protein kinase MPK3 [47]. In this study, we showed that expression of IrrE in *E. coli* and the plant *Brassica napus* results in increased tolerance to a number of stresses, particularly salt stress. We cannot discount the possibility that IrrE may have an as yet undiscovered role and that its expression in *E. coli* and *B. napus* cells promotes functions contributing to salt tolerance. But results from our proteome analysis favor the notion that IrrE acts as a global regulator to regulate different sets of proteins such as stress-responsive proteins, protein kinases, glycerol-degrading enzymes, detoxification proteins and growth-related proteins in *E. coli*. It is well known that salt-stress also causes oxidative damage to cells as it induces ROS accumulation and that oxidative damage protection is an important aspect of salt-stress tolerance [2]. We also observed a significant induction of several detoxification proteins in IrrE-expressing *E. coli*, including a catalase HPII (KatE), which serves to protect cells from the toxic effects of hydrogen peroxide, and a tryptophan repressor binding protein (WrbA) which could play a role in the oxidative stress response of diverse prokaryotes. Quantitative RT-PCR analysis revealed that genes encoding manganese superoxide dismutase SOD were upregulated in transgenic *Brassica napus* plants, which also suggests the existence of similar regulatory systems of IrrE in both *E. coli* and *B. napus*. Therefore, changes in the levels of detoxification proteins and stress-responsive genes may collectively contribute to the increased salt tolerance in these genetically modified organisms. Further studies are required to identify its direct target genes or its interacting partners in order to elucidate the molecular mechanisms of IrrE in conferring improved salt tolerance in plants. Our results presented here may provide new insights into the molecular basis of IrrE functions contributing to salt tolerance, and demonstrate its utility as an effective means of manipulating abiotic stress tolerance in crop plants.

## Materials and Methods

### Expression of the *irrE* gene in *E. coli* JM109

The GroESL promoter [48] was amplified from *D. radiodurans* R1 genomic DNA by PCR with primers 5′-GGATACCCCCATTCCCCGTCCCAGC-3′ and 5′-CCGCGGTCGCCTAAAGGTTTCAGCATATGG-3′. The PCR product was ligated into the T-cloning site of T-vector pMD18T (TaKaRa Bio Dalian CO., LTD), generating the plasmid pMG1. The *irrE* gene was amplified from *D. radiodurans* R1 genomic DNA by PCR with the following primers 5′-GGAATTCCATATGTGCCCAGTGCCAACG-3′ and 5′-TCCCCGCGGAGATCTCCAGTTCACTGTG-3′. The PCR product was digested with *Nde* I and *Sac* II and ligated into the *Nde* I and *Sac* II sites of pMG1, generating the plasmid pMG1-IrrE. Plasmid pMG1-IrrE was used for expression of the *irrE* gene under control of a GroESL promoter in *E. coli* JM109, and plasmid pMG1 was used as the empty plasmid control.

### Overexpression of the *irrE* gene in transgenic *B. napus* plants

The coding region of the *irrE* gene was amplified from genomic DNA of *D. radiodurans* R1 using primers 5′-TGCTCTAGAATGTGCCCAGTGCCAAC-3′ and 5′-CGAGCTCCCAGTTCACTGTGCAGC-3′. The PCR product was digested with *Xba* I and *Sac* I, and inserted into the *Xba* I and *Sac* I sites of the pBI121 binary vector between the CaMV35S promoter and the nopaline synthase terminator [49]. The resulting vector pBI-IrrE (**Figure 2A**) was introduced into *Agrobacterium tumefaciens* strain EHA105 by triparental mating. *Brassica napus* cultivar Shuanzha no.9 was transformed as described [19]. When the green shoots were 1–2 cm tall, they were separated from the calli and transferred onto rooting medium supplemented with 75 mg/l kanamycin and 200 mg/l ampicillin. Under these conditions, about 87% of the shoots formed roots in two weeks. Rooted shoots were transplanted to soil and seeds (T1) were collected. T1 seeds were grown on modified Murashige and Skoog medium plates supplemented with 75mg/l kanamycin and 200 mg/l ampicillin to select homozygous T2 transgenic lines.

### Abiotic stress-resistance assays

The control strain carrying only vector pMG1 and the transformant strain expressing IrrE were grown in LB medium at 37°C until the OD_600_ reached 0.5. Cells were then pelleted from 1 ml cultures by centrifugation to remove the growth medium before transferring to fresh LB medium in the presence or absence of 3.0 M NaCl, 3.0 M sorbitol and 1% H_2_O_2_. Cell suspensions (OD_600_ approximately 0.5) were incubated at 37°C at different times. For heat stress, cell suspensions (OD_600_ approximately 0.5) were incubated at 52°C for 1 h. After incubation, serial dilutions of 10 times were made. Ten microliters of each dilution was spotted onto LB solid plates. For growth assays under salt stress, *E. coli* strains were grown in LB medium until the stationary phase was achieved and then sub-cultured in M9 minimal medium [50] until an OD_600_ of 0.5 was reached. Cultures were then diluted to an OD_600_ of 0.1 before inoculating fresh M9 minimal medium containing 0.65 M NaCl or unsupplemented medium. Growth rate was monitored by absorbance change at 600 nm. Data are the means±SD of three independent experiments with three replicates per experiment. For greenhouse salt-tolerance experiments, wild-type and transgenic seeds (T2) were germinated and grown in the greenhouse according to a published protocol [19]. Two weeks after germination, plants were treated with NaCl at 350 mM for six weeks in pots, and then transferred to the field.

### Proteomic analysis

The *E. coli* control strain carrying only vector pMG1 and the transformant strain expressing IrrE, respectively, were grown with shaking at 37°C in LB medium until the cultures reached an OD_600_ of 0.4. Subsequently, NaCl was added to the medium (final concentration 1 M) and cells were harvested after 1 h. After centrifugation at 10,000 *g* for 10 min, cellular proteins from the supernatant were collected and cellular proteins from the pellet were solubilized. The protein concentration was measured using the Bradford method [51]. The first dimension (IEF) was carried out on IPG strips (Amersham Pharmacia Biotech, Uppsala, Sweden) in a Multiphor II electrophoresis unit (Amersham Pharmacia Biotech). For the second dimension, vertical slab SDS/PAGE (12%) was run in a Bio-Rad Protean II Xi unit (Bio-Rad Laboratories, Hercules, CA). Gels were stained with colloidal CBB G-250, and scanned with a PowerLook 1000 (UMAX Technologies Inc., Dallas, TX). PDQuest version 7.3.0 (Bio-Rad Laboratories) was used for image analysis. For MALDI-TOF MS analysis, protein spots were excised from gels, digested with trypsin as described [52], and the various peptides generated were analyzed using a 4700 Proteomics Analyzer (Applied Biosystems). Proteins were identified by automated peptide mass fingerprinting using the Global Proteome Server Explorer software (Applied Biosystems).

### Quantitative RT-PCR analysis

Total RNA of transgenic and wild-type plants were extracted from the leaves of 2-week-old plants treated with 350 mM NaCl for 24 h using TRIzol reagent (GIBCO-BRL). RNase-free DNase I (TaKaRa Bio Dalian CO., Ltd) was used to digest the genomic DNA in the total RNA preparation. Approximately one hundred micrograms of total RNA was then synthesized into cDNA using a Reverse-Transcription Kit (TaKaRa Bio Dalian CO., Ltd). The expression levels of the selected genes were determined using the iCycler iQ Real-time PCR Detection System (Bio-Rad) according to the manual of Two-step QuantiTect SYBR Green PCR Kit. Data processing was done by iCycler real-time detection system software (version 2.0). The primers used for qRT-PCR amplifications of the ten selected genes are described in **Table S3**. Expression levels were normalized using values obtained for the housekeeping gene β-actin and then normalized to wild-type levels. All real-time PCRs were performed in duplicate in three independent experiments.

### Assays of intercellular glycerol content

Cells were cultured at 37°C in LB medium and harvested at the early exponential phase (OD_600_ between 0.4–0.5). Subsequently, cells were resuspended in fresh medium with or without 1.0 M NaCl. After incubation at 37°C for 1 h, cells were harvested and prepared as described [53]. The glycerol content was assayed using the glycerol GPO assay kit (Beijing SIONPCR Biological Engineering Ltd. Co.). Data represent the means and standard errors of three replicate experiments.

### Protein isolation and western blots

Protein isolation from four-week-old plants was performed as described [54]. Protein content was determined by Bio-Rad assay. Western blots of the different membrane fractions were performed as described [55].

## Supporting Information

Table S1 Upregulated proteins of the E. coli strain expressing IrrE versus the control strain carrying only the pMG1vector in response to salt shock
(0.14 MB DOC)

Table S2 Downregulated proteins of the E. coli strain expressing IrrE versus the control strain carrying only the pMG1vector in response to salt shock
(0.13 MB DOC)

Table S3 Primers used for qRT-PCR amplifications in this study
(0.04 MB DOC)
