# Supplementary material for: IrrE, a Global Regulator of Extreme Radiation Resistance in Deinococcus radiodurans, Enhances Salt Tolerance in Escherichia coli and Brassica napus
Source: PLoS One. 2009 Feb 10;4(2):e4422. doi: 10.1371/journal.pone.0004422 (PMC2635966; doi:10.1371/journal.pone.0004422)
Supplement: Table S1 — Upregulated proteins of the E. coli strain expressing IrrE versus the control strain carrying only the pMG1vector in response to salt shock (0.14 MB DOC) [file pone.0004422.s001.doc]

**Table S1** Upregulated proteins of the *E. coli* strain expressing IrrE versus the control strain carrying only the pMG1vector in response to salt shock

| Protein^a^ | Description | pI/Mr (kDa) | | Induction ratio^b^ |
| --- | --- | --- | --- | --- |
|  |  | Theoretical | Experimental |  |
| *Biosynthesis of small molecules* | |  |  |  |
| Nucleotides | |  |  |  |
| Ndk | Nucleoside diphosphate kinase | 5.54/15.42 | 5.87/14.66 | 3.13 |
| PurB | Adenylosuccinate lyase | 5.68/51.48 | 6.32/46.27 | Present^c^ |
| PurC | Phosphoribosylaminoimidazole-succinocarboxamide synthase | 5.16/27.34 | 5.02/29.04 | 5.64 |
| PurD | Phosphoribosylglycineamide synthetase | 4.96/45.91 | 5.10/43.75 | Present |
| PurE | Phosphoribosylaminoimidazole carboxylase catalytic subunit | 6.58/18.80 | 6.68/17.31 | Present |
| PurF | Amidophosphoribosyltransferase | 5.39/56.53 | 5.35/63.57 | Present |
| PurH | Phosphoribosylaminoimidazolecarboxamide formyltransferase and fused IMP cyclohydrolase | 5.53/57.29 | 5.87/52.40 | 18.94 |
| PurK | Phosphoribosylaminoimidazole carboxylase ATPase subunit | 5.50/39.53 | 5.93/38.07 | Present |
| PurL | Phosphoribosylformyl-glycineamide synthetase | 5.23/141.31 | 5.37/118 | Present |
| PurT | Phosphoribosylglycinamide formyltransferase 2 | 5.48/42.40 | 5.70/42.27 | Present |
| PyrB | Aspartate carbamoyltransferase catalytic chain | 6.12/34.40 | 6.62/35.38 | 5.32 |
| PyrC | Dihydro-orotase | 5.77/38.80 | 6.10/39.18 | 4.63 |
| CarA | Carbamoyl-phosphate synthase small chain | 6.00/41.40 | 6.31/42.56 | 2.34 |
| CarB | Carbamoyl-phosphate synthase large subunit | 5.22/117.76 | 5.35/116.05 | 4.51 |
| Amino acids | |  |  |  |
| AsnB | Asparagine synthetase B | 5.55/62.61 | 5.71/64.58 | 12.22 |
| GltD | Glutamate synthase, small subunit | 5.54/51.98 | 5.72/57.05 | 2.41 |
| GlyA | Serine hydroxymethyltransferase | 6.03/45.27 | 6.49/44.93 | 4.07 |
| PheA | Chorismate mutase-P/prephenate dehydratase | 6.21/43.02 | 5.69/93.45 | 15.10 |
| SerA | D-3-phosphoglycerate dehydrogenase | 5.92/44.14 | 6.36/44.49 | 2.56 |
| SerC | 3-phosphoserine aminotransferase | 5.49/39.82 | 5.51/39.03 | 2.90 |
| TyrA | Chorismate mutase T/prephenate dehydrogenase | 5.58/41.98 | 5.97/39.94 | 4.09 |
| Cofactors, small molecule carriers | |  |  |  |
| FolD | 5,10-methylene-tetrahydrofolate dehydrogenase / 5,10-methylene-tetrahydrofolate cyclohydrolase | 5.91/30.93 | 6.22/33.81 | 2.66 |
| NadC | Quinolinate phosphoribosyltransferase | 5.07/32.75 | 5.30/38.53 | Present |
| PanC | Pantothenate synthetase | 5.91/31.57 | 6.25/33.30 | 2.09 |
| MenB | Naphthoate synthase | 6.00/31.62 | 6.67/34.21 | 3.45 |
| *Macromolecule metabolism* | |  |  |  |
| Ribosomal proteins | |  |  |  |
| RpsF | 30S ribosomal subunit protein S6 | 5.26/15.17 | 5.43/15.14 | 22.66 |
| RpsA | 30S ribosomal protein S1 | 4.89/61.12 | 4.84/76.43 | 2.44 |
| RNA synthesis,modification,DNA transcription | |  |  |  |
| Rnb | Exoribonuclease II | 5.45/72.38 | 5.67/85.13 | 4.14 |
| RsuA | 16S rRNA pseudouridylate 516 synthase | 5.75/25.84 | 6.14/32.41 | 2.20 |
| AsnC | DNA-binding transcriptional regulator | 6.29/16.87 | 6.4514.10 | Present |
| PspA | Phage shock protein, putative inner membrane protein | 5.51/25.56 | 5.50/29.00 | 5.83 |
| RpoS | RNA polymerase sigma factor S variant | 4.89/37.93 | 4.93/41.26 | 3.87 |
| Proteins (translation and modification) | |  |  |  |
| PheS | Phenylalanine tRNA synthetase, alpha subunit | 5.79/36.80 | 6.38/36.72 | 4.20 |
| PheT | Phenylalanine tRNA synthetase, beta subunit | 5.17/87.32 | 5.27/97.79 | Present |
| SelD | Selenophosphate synthase | 5.29/36.64 | 5.52/38.51 | Present |
| Frr | Nibosome releasing factor | 6.43/19.21 | 6.88/24.70 | 2.21 |
| Lon | ATP-dependent protease La | 6.01/87.38 | 6.40/97.53 | Present |
| Proteins (chaperones) | |  |  |  |
| DnaK | Chaperone Hsp70 | 4.83/69.07 | 4.75/79.36 | 6.09 |
| HslU | Heat shock protein | 5.24/48.56 | 5.32/50.85 | 3.03 |
| Dps | Stress response DNA-binding protein | 5.72/18.68 | 6.30/18.51 | 2.52 |
| PpiB | Ppeptidyl-prolyl cis-trans isomerase B | 5.52/18.15 | 5.62/16.60 | Present |
| Tig | Trigger factor | 4.73/47.99 | 4.84/55.28 | Present |
| Degradation of macromolecules | |  |  |  |
| OmpT | Naphthoate synthase | 5.76/35.55 | 5.52/37.55 | 2.92 |
| *Energy metabolism* | |  |  |  |
| TCA cycle | |  |  |  |
| Ppc | Phosphoenolpyruvate carboxylase | 5.52/99.01 | 5.72/98.77 | 2.96 |
| Pentose phosphate pathway | |  |  |  |
| Eda | KHG/KDPG aldolase | 5.57/22.26 | 5.73/25.38 | 2.61 |
| TktB | Transketolase 2, thiamin-binding | 5.86/72.99 | 6.37/83.36 | 2.61 |
| TalA | Transaldolase A | 5.89/35.63 | 6.27/38.06 | 2.00 |
| *Carbon utilization* | |  |  |  |
| DhaH | Fused predicted dihydroxyacetone-specific PTS enzyme HPr component and EI component | 4.61/51.41 | 4.61/49.06 | Present |
| GcvP | Glycine dehydrogenase | 5.62/104.28 | 5.90/100.28 | 2.63 |
| GcvT | Aminomethyltransferase | 5.36/40.12 | 5.70/38.99 | 3.51 |
| KdgK | Ketodeoxygluconokinase | 4.92/33.94 | 4.92/37.90 | 7.81 |
| KduI | 4-deoxy-L-threo-5-hexosulose-uronate ketol-isomerase | 5.70/31.05 | 6.04/31.28 | Present |
| PoxB | Pyruvate oxidase | 5.81/60.85 | 6.31/68.91 | 3.00 |
| *Central intermediary metabolism* | |  |  |  |
| KduD | 2-deoxy-D-gluconate 3-dehydrogenase | 5.64/27.03 | 5.43/27.07 | 2.69 |
| *Transporters* | |  |  |  |
| Channel-type Transporters | |  |  |  |
| AtpD | ATP synthase beta subunit | 4.89/46.19 | 4.87/49.63 | 3.98 |
| OmpF | Outer membrane protein F precursor | 4.78/39.33 | 4.62/37.60 | Present |
| oppA | oligopeptide transport; periplasmic binding protein | 5.95/60.98 | 6.38/62.03 | Present |
| PotA | ATP-binding component of spermidine / putrescine transport | 5.19/43.02 | 5.34/42.86 | 3.14 |
| *Cytoskeleton* | |  |  |  |
| GlmS | D-fructose-6-phosphate amidotransferase | 5.61/66.80 | 5.75/76.76 | 2.04 |
| RmlA | Glucose-1-phosphate thymidylyltransferase | 5.39/32.67 | 5.69/31.90 | 2.43 |
| YeaF | MltA-interacting protein precursor | 5.50/27.81 | 4.94/28.29 | 2.98 |
| *Cellular processes* | |  |  |  |
| Adaptation | |  |  |  |
| OsmY | Osmotically inducible protein Y precursor | 6.75/24.05 | 5.45/20.86 | 4.41 |
| YhbO | Putative intracellular proteinase | 5.27/18.84 | 5.47/23.02 | Present |
| Protection | |  |  |  |
| KatE | hydroperoxidase HPII | 5.57/84.11 | 5.80/95.30 | 4.34 |
| WrbA | Trp repressor binding protein; affects association of trp repressor and operator | 5.67/18.82 | 5.91/24.40 | 3.58 |
| *Hypothetical,unclassified,or unknown* | |  |  |  |
| YgaU | Conserved hypothetical protein | 5.71/16.02 | 6.19/16.46 | 2.92 |

^a^Protein names, accession numbers, and descriptions are from ExPASy Server (<http://kr.expasy.org/>). The data are grouped according to their biological function.

^b^Induction ratio (cells expressing IrrE/cells carrying only vector pMG1) after 60 min, with 1.0 M NaCl.

^c^Present: all of these proteins were detectable only in the IrrE-expressing strain, and they were absent in the control strain.
