# Supplementary material for: IrrE, a Global Regulator of Extreme Radiation Resistance in Deinococcus radiodurans, Enhances Salt Tolerance in Escherichia coli and Brassica napus
Source: PLoS One. 2009 Feb 10;4(2):e4422. doi: 10.1371/journal.pone.0004422 (PMC2635966; doi:10.1371/journal.pone.0004422)
Supplement: Table S2 — Downregulated proteins of the E. coli strain expressing IrrE versus the control strain carrying only the pMG1vector in response to salt shock (0.13 MB DOC) [file pone.0004422.s002.doc]

**Table S2** Downregulated proteins of the *E. coli* strain expressing IrrE versus the control strain carrying only the pMG1vector in response to salt shock

| Protein^a^ | Description | pI/Mr (kDa) | | Induction ratio^b^ |
| --- | --- | --- | --- | --- |
|  |  | Theoretical | Experimental |  |
| *Biosynthesis of small molecules* | |  |  |  |
| Amino acids | |  |  |  |
| AroD | 3-dehydroquinate dehydratase | 5.19/27.46 | 5.37/29.44 | 0.38 |
| AspA | Aspartate ammonia-lyase | 5.54/54.02 | 5.23/41.79 | 0.39 |
| MetL | AKII-HDII protein | 5.33/88.84 | 5.52/93.97 | 0.17 |
| Cofactors, small molecule carriers | |  |  |  |
| HemB | Porphobilinogen synthase | 5.25/35.60 | 5.37/38.76 | 0.31 |
| NfsA | Nitroreductase A, NADPH-dependent, FMN-dependent | 6.45/26.78 | 6.85/26.90 | 0.22 |
| *Macromolecule metabolism* | |  |  |  |
| Ribosomal proteins | |  |  |  |
| RpsB | 30S ribosomal subunit protein S2 | 6.61/26.72 | 4.21/31.80 | 0.28 |
| RplU | 50S ribosomal subunit protein L21 | 9.85/11.57 | 7.00/13.75 | 0.24 |
| RNA synthesis,modification,DNA transcription | |  |  |  |
| RpoC | DNA-directed RNA polymerase beta' chain | 6.67/155.05 | 5.78/84.17 | 0.45 |
| LacI | lac repressor | 6.39/38.56 | 6.85/39.80 | Absent^c^ |
| Pnp | Polynucleotide phosphorilase | 5.15/77.12 | 5.24/55.46 | 0.21 |
| KdgR | Transcriptional regulator KdgR | 5.31/30.01 | 5.72/32.16 | Absent |
| PdhR | Transcriptional regulator for pyruvate dehydrogenase complex | 6.04/29.40 | 6.60/31.15 | 0.44 |
| PurR | Purine nucleotide synthesis repressor | 6.47/38.13 | 6.76/37.85 | Absent |
| Proteins (translation and modification) | |  |  |  |
| TufA | Translation elongation factor EF-Tu | 5.25/44.85 | 5.47/47.51 | Absent |
| Proteins (chaperones) | |  |  |  |
| HslV | ATP-dependent hslVU protease peptidase subunit hslV | 5.96/19.09 | 6.31/22.34 | 0.22 |
| GroE | Chaperone Hsp60, peptide-dependent ATPase, heat shock protein | 4.81/57.27 | 4.84/63.55 | 0.26 |
| RdoA | Predicted kinase | 4.99/38.09 | 5.14/37.99 | 0.02 |
| *Energy metabolism* | |  |  |  |
| TCA cycle | |  |  |  |
| FumA | Fumarate hydratase Class I | 6.11/60.25 | 6.64/55.82 | 0.49 |
| Glycolysis/gluconeogenesis | |  |  |  |
| GapA | Glyceraldehyde 3-phosphate dehydrogenase A | 6.33/35.93 | 6.78/36.77 | 0.32 |
| MaeB | Putative multimodular enzyme | 5.34/82.42 | 5.58/90.80 | 0.45 |
| PckA | Phosphoenolpyruvate carboxykinase | 5.73/62.39 | 5.56/64.55 | 0.31 |
| LpdA | Dihydrolipoamide dehydrogenase | 6.01/53.09 | 6.17/48.03 | 0.49 |
| Anaerobic respiration | |  |  |  |
| AceE | Pyruvate dehydrogenase E1 component | 5.46/99.60 | 5.50/103.20 | 0.44 |
| FrdA | Fumarate reductase, anaerobic, flavoprotein subunit | 5.86/65.92 | 6.10/80.48 | 0.35 |
| NarH | Nitrate reductase beta subunit | 6.36/58.02 | 6.89/68.08 | 0.16 |
| PflD | Putative formate acetyltransferase 3 | 5.58/85.88 | 5.76/87.04 | 0.24 |
| TdcE | Pyruvate formate-lyase 4/2-ketobutyrate formate-lyase | 5.48/85.88 | 5.76/92.11 | 0.11 |
| Fermentation | |  |  |  |
| AdhE | PFL-deactivase / alcohol dehydrogenase / acetaldehyde dehydrogenase | 6.25/96.12 | 6.79/89.74 | 0.19 |
| *Carbon utilization* | |  |  |  |
| AckA | Acetate kinase | 5.85/43.26 | 6.08/41.98 | 0.49 |
| AldA | Aldehyde dehydrogenase, NAD-linked | 5.07/52.20 | 5.08/56.63 | 0.42 |
| GarR | Tartronate semialdehyde reductase | 5.58/30.63 | 5.87/31.87 | 0.13 |
| GatY | Tagatose-bisphosphate aldolase GatY | 5.98/30.96 | 6.38/31.20 | 0.28 |
| GatZ | Tagatose-1,6-bisphosphate aldolase 2 | 5.50/42.31 | 5.70/47.96 | 0.35 |
| TreC | Trehalose-6-P hydrolase | 5.51/63.79 | 5.70/68.45 | Absent |
| TnaA | Tryptophanase/L-cysteine desulfhydrase, PLP-dependent | 5.88/52.73 | 6.07/49.57 | 0.12 |
| UxuA | Mannonate dehydratase | 5.39/44.79 | 5.68/40.39 | 0.09 |
| GldA | Glycerol dehydrogenase | 4.81/38.68 | 4.78/39.91 | 0.46 |
| GlpA | Anaerobic glycerol-3-phosphate dehydrogenase subunit A | 6.14/58.86 | 6.64/69.32 | 0.28 |
| GlpB | Sn-glycerol-3-phosphate dehydrogenase (anaerobic), membrane anchor subunit | 5.75/45.32 | 6.12/41.43 | 0.26 |
| GlpK | Glycerol kinase | 5.36/56.19 | 5.50/54.04 | 0.44 |
| *Central intermediary metabolism* | |  |  |  |
| Hmp | Dihydropteridine reductase | 5.48/43.81 | 5.72/41.28 | 0.17 |
| TdcD | Propionate kinase/acetate kinase C, anaerobic | 5.63/43.90 | 5.68/40.53 | Absent |
| YghU | Predicted S-transferase | 6.21/32.37 | 6.66/33.96 | 0.22 |
| *Transporters* | |  |  |  |
| Channel-type Transporters | |  |  |  |
| LamB | Phage lambda receptor protein; maltose high-affinity receptor | 4.72/47.35 | 4.75/43.08 | 0.44 |
| MalK | Maltose ABC transporter | 6.77/40.88 | 6.93/61.30 | 0.33 |
| MglB | Galactose ABC transporter | 5.53/87.81 | 5.20/34.30 | Absent |
| OmpP | Outer membrane proease P | 5.91/35.47 | 6.29/34.70 | Absent |
| RbsB | D-ribose periplasmic binding protein | 5.85/30.91 | 6.32/30.01 | 0.22 |
| YrbF | Putative toluene transporter subunit: ATP-binding component | 6.16/29.07 | 6.77/31.48 | 0.20 |
| *Cytoskeleton* | |  |  |  |
| GalE | UDP-galactose-4-epimerase | 5.89/37.21 | 6.42/37.73 | 0.26 |
| *Cellular processes* | |  |  |  |
| Adaptation | |  |  |  |
| YfiD | Stress-induced alternate pyruvate formate-lyase subunit | 5.58/14.97 | 5.05/13.43 | 0.47 |
| Protection | |  |  |  |
| MdaB | Modulator of drug activity B | 5.84/21.86 | 6.26/24.03 | 0.18 |
| NfnB | Dihydropteridine reductase, NAD(P)H-dependent, oxygen-insensitive | 5.50/23.97 | 6.37/26.49 | 0.30 |
| MutS | MutHLS complex, methyl-directed mismatch repair | 5.51/91.32 | 5.59/68.66 | 0.41 |
| *Hypothetical,unclassified,or unknown* | |  |  |  |
| YcaO | Conserved hypothetical protein | 4.38/65.61 | 4.48/57.95 | Absent |
| YeiR | Hypothetical protein | 6.00/36.09 | 6.37/35.92 | Absent |
| YjtD | Putative ATP synthase beta subunit | 5.82/26.90 | 6.09/29.03 | 0.28 |
| YqhD | Putative alcohol dehydrogenase, NAD(P)-dependent | 5.72/42.06 | 6.13/38.95 | 0.31 |

^a^Protein names, accession numbers, and descriptions are from ExPASy Server (<http://kr.expasy.org/>). The data are grouped according to their biological function.

^b^Induction ratio (cells expressing IrrE/cells carrying only vector pMG1) after 60 min, with 1.0 M NaCl.

^c^Absent: all of these proteins were absent in the IrrE-expressing strain, and they were detectable only in the control strain.
