# Supplementary material for: IrrE, a Global Regulator of Extreme Radiation Resistance in Deinococcus radiodurans, Enhances Salt Tolerance in Escherichia coli and Brassica napus
Source: PLoS One. 2009 Feb 10;4(2):e4422. doi: 10.1371/journal.pone.0004422 (PMC2635966; doi:10.1371/journal.pone.0004422)
Supplement: Table S3 — Primers used for qRT-PCR amplifications in this study (0.04 MB DOC) [file pone.0004422.s003.doc]

| Gene | Forward Primer | Reverse Primer |
| --- | --- | --- |
| β-Actin | ACTGTGCCAATCTACGAGGGTT | TCTTACAATTTCCCGCTCTGCT |
| IrrE | ACGCTGGCCCAAGCACAGAAA | CGTCCACCTCCGCCTTCATTTT |
| CBF1 | CAAATCCGCCTGCCTCAA | CATCGCTGTCTTCCTCCGTAA |
| CBF3 | AGTGAGGGAACCAAACAAGAAA | CAAGCCGAGTCCGCATAAT |
| SOS1 | AAGGGGAGTCGCTGATGAATG | GAGCCAAAGAACCGAGACAATG |
| SOS2 | GGCAGTTATGTAGCGGAGAAT | GCCTGTCGCCTGTCAAATAGT |
| CAT | GAAGGTTTCGGCGTCCACA | GAAGGTTTCGGCGTCCACA |
| SOD | GTTCAACGGCGGAGGTCA | AACATCAATACCCACCAGAGGA |
| HSP | CTCTTGCGTCGGAGTATGGC | AAGACGGCGTGGTCCTGTTA |
| PIP | GATAACGGCTGCTCCGAAACTC | TTCTCCGCCACTGACCCTAAAC |
| PP2A | CTCAAGCAAGGCTCCACATACG | GGCAGAAGACAGGAAACGAAGT |

**Supplementary Table 3** Primers used for qRT-PCR amplifications in this study

Abbreviations: CBF1/ CBF3, CBF-like protein; SOS2, serine/threonine protein kinase; SOS1, the plasma membrane Na^+^/H^+^ antiporter; CAT, catalase; SOD, manganese superoxide dismutase; HSP, heat shock cognate protein Hsc70; PIP, the plasma membrane intrinsic proteins PIPs; and PP2A, phosphatase 1.
